# Mindfulness-Based Approaches for COVID-19 Mental Health in Working from Home

**DOI:** 10.1007/s11469-021-00647-3

**Published:** 2021-11-03

**Authors:** Katia C. Vione, Yasuhiro Kotera

**Affiliations:** School of Psychology, University of Derby, DE22 1GB, Derbyshire, UK

**Keywords:** Mindfulness, Compassion, Remote working, COVID-19, Mental health

## Abstract

The novel coronavirus (COVID-19) pandemic spread rapidly since it was first identified in December 2019. A nation-level lockdown has been implemented in many countries, affecting workers of all sectors and forcing many to work from home. In this commentary, we discuss mental health difficulties that working from home might cause on non-key workers, based on research in New Ways of Working (NWW) and telecommuting. Moreover, we propose the use of mindfulness-based approaches to protect workforce from the potential negative impacts of working from home.

Since the first case was identified in the province of Hubei, China in December 2019, the novel coronavirus disease 2019 (COVID-19) has spread worldwide, and currently (as of 1^st^ September 2021), over 210 million cases have been confirmed. To prevent its rapid infection, a nation-level lockdown has been implemented in many countries, impacting people’s way of living, including their ways of working. Workers in many countries were forced to adjust to more flexible and adaptive work styles, similar to what has been described in the literature as New Ways of Working (NWW; Slagter, 2011). NWW is a relatively new trend where organizations transform work relationships to provide flexibility in terms of time and space. In other words, working tasks can be carried out at varied times and outside of the office, including from workers’ own home. This is usually supported by the active use of information and communications technology (ICT) and clearly defined targets. Companies that transition into NWW normally have time to prepare and implement the transformation, but still negative psychological impacts have been reported in the literature (Fedakova & Ištoňová, 2017; Kotera & Vione, 2020; Van Steenbergen et al., 2018). In the COVID-19 pandemic, the transformations in people’s way of working came unannounced; however, we can use our understanding of the impacts of NWW to propose psychological interventions to counter the negative impacts of working from home. In this commentary, we will discuss the current mental health difficulties of workers impacted by COVID-19 and possible psychological interventions to protect workforce from these potential negative impacts.

## Mental Health Difficulties for Workers During COVID-19

The COVID-19 pandemic has received considerable attention from researchers across the globe. For example, The Lancet has launched the COVID-19 Resource Center (2020) to archive articles and news that are freely accessible online. A social media website for researchers, ResearchGate has created the COVID-19 Community (2021), which has so far computed over 300,000 outputs, including empirical studies, commentaries and recommendation articles. One area of increasing focus in the COVID-19 research communities is placed on mental health (Martin & Cooper, 2020). As identified in the SARS outbreak, pandemics can impact mental health negatively such as heightened levels of depression and stress, especially in those who were isolated (Ko et al., 2006).

Similarly, in the current pandemic, Lei et al. (2020) investigated the prevalence of anxiety and depression in 1593 participants from China in February 2020, comparing those who had been affected by quarantine (whether they or their families/colleagues/classmates/neighbors had been quarantined) to those unaffected. The prevalence of anxiety and depression in the unaffected group was approximately 7% and 12%, respectively, while the prevalence of these mental health issues in the affected group was 13% for anxiety and 22% for depression: almost twice higher than the unaffected. Thus, those who had been affected by quarantine had significantly poorer mental health than those who were not affected. Additionally, another study investigated the prevalence of Post-Traumatic Stress Disorder (PTSD) in the hardest hit areas in China 1 month after the outbreak. The researchers reported a prevalence of 7% of PTSD among 285 Wuhan residents (Liu et al., 2020). Arslan et al. (2020) observed that stress associated with the coronavirus spread significantly affected people’s level of optimism, psychological inflexibility, and psychological problems (e.g., depression, anxiety, and somatization). These findings of different studies during the coronavirus pandemic support that lockdown measures, including quarantine, affect people’s mental health and the implications can vary among different groups.

Among different population groups, mental health of healthcare workers (HCW) has been evaluated, these workers face direct risks to their own health, risk of exposing their families to the virus, increased stress, burnout, and fatigue (Koh & Goh, 2020). In many countries, HCW also had to face difficult situations such as loneliness, uncertainty, and moral challenges (e.g., having to choose who to save, due to lack of resources to provide adequate care to all patients) (Schwartz et al., 2020).

Other key workers have also been the focus of concern but the impact of the pandemic on the remaining of the world workforce has been mostly neglected by the academic community (Shimazu et al., 2020). For most of the workers, they had to rapidly adapt to the changes associated with COVID-19 including working from home, sharing the responsibility of maintaining businesses and guaranteeing their own job security. Unsurprisingly, high levels of depression, anxiety, and stress in workers from other sectors than healthcare have been reported (Arafa et al., 2020; Bouziri et al., 2020).

In summary, current findings from the COVID-19 pandemic and findings from the SARS outbreak provide clear evidence of negative mental health impacts for the occupational populations, including HCW and other workers. It is also evident that these groups differ in terms of how their mental health is affected, but little is known about how working from home, in this context, has affected workers’ mental health.

## Psychological Impacts of Working from Home

As previously mentioned, literature about NWW and telecommuting can help us understand the psychological impacts that working from home might cause. There are three distinguishing features of NWW: (1) workers can choose when they work, (2) where they work, and (3) work is facilitated by information technology (Van Steenbergen et al., 2018). During the COVID-19 lockdown, workers had limited choice of places they could work, being restricted to work from home, combining family and workspace in the same household, sometimes with more than one worker. Still, companies had to quickly adapt to allow time flexibility and implement technologies to allow businesses to function with staff working remotely. The current situation is also similar to the concept of telecommuting—work carried out remotely where the worker communicates with co-workers using technologies (Di Martino & Wirth, 1990). Research on these modes of working remotely has provided evidence about the psychological impacts of working from home.

In terms of positive impacts of working from home, the associated benefits of reduced commuting time and costs, reduced environmental pollution, and opportunity to support family duties (e.g., picking up children from school) might be desirable for many workers. There are also psychological advantages of this mode of work, such as increase in engagement, enjoyment, and connectivity (Kotera & Vione, 2020), especially in the presence of positive communication and social interactions (e.g., Gerards et al., 2018, Ten Brummelhuis et al., 2012). Peters et al. (2014) observed that employees who worked from home at least 1 day per week, demonstrated higher autonomy, achieving higher levels of flow (enjoyment, absorption, and intrinsic motivation), which was also improved by a perception of supervisor and collegial support. Therefore, remote working can have positive psychological effects on workers, and this is partly supported by their perception of support from supervisors and colleagues.

Perhaps the most intuitive negative effect of working from home is the compromise of the relationship with other colleagues. Gajendran and Harrison’s (2007) meta-analysis identified that telecommuters that worked from home more than 2.5 days a week had less positive relationships with their colleagues. Working from home also affects the relationships with family. Fedakova and Ištoňová, (2017) observed that NWW blurred psychological boundaries between work and family. In this study, although the companies offered flexibility to employees to work from home, they were still required to adhere to the companies’ standard work schedules. This reduced flexibility reflects the reality of many employees who had to start working from home during the COVID-19 pandemic. Therefore, it is important to provide special attention to the effects of blurred boundaries between work and family, considering difficulties that working from home might bring about, in terms of switching patterns of emotions and behaviors that previously would be expressed only in the workplace, defining the line between work and family (Clark, 2000).

Additionally, other psychological effects of NWW were reported in Kotera and Vione’s (2020) review, which indicated that this remote way of working increased fatigue, mental demands, and blurred work-family boundaries. Van Steenbergen et al. (2018) conducted a study with employees from a company going through an NWW transformation. Data from three time points, including before and after the transformation, indicated that job resources were more severely impacted by this type of work than the conventional working from office. Employees who work from home report that they perceive less opportunities for professional development. Surprisingly, this study also indicated a reduction in autonomy, which could be understood as a lack of choice regarding the transformation and not necessarily about how employees do their work. However, the reasons for this change in perception was not part of the study. Similarly, workers did not have a choice during the COVID-19 lockdown; thus, it is possible that it has impacted workers perception of autonomy—a key factor for wellbeing and work-life balance (Fernet et al., 2014).

Other negative outcomes have also been observed when comparing teleworkers and office workers. Mann and Holdsworth (2003) conducted a mixed-methods study with journalists, in which they first interviewed office workers and telecommuters, then, conducted a survey in a separate sample of workers in the same work conditions. Results from the interviews indicated that teleworkers experienced more negative emotions of loneliness, irritability, worry, and guilt. These were then corroborated with survey results, where teleworkers presented higher levels of stress than office workers.

In summary, although there are associated benefits of working from home, these are dependent on organizational and social support. Thus, it is important to focus on prevention and mitigation of the negative effects on worker’s mental health: working from home appears to increase levels of loneliness, irritability, worry, guilt, and stress. Moreover, it can compromise family relationships and, in some cases, reduce the sense of autonomy. These issues are likely to be affecting employees working from home during the pandemic: the occupational health researchers must propose potential solution to counter these negative impacts.

## Mindfulness-Based Approaches for COVID-19 Mental Health

Based on the issues discussed, psychological interventions to help workers during this period should target stress, worry, and loneliness. Particularly loneliness bears greater importance as an increased sense of loneliness is regarded as a global human phenomenon today, leading to diverse psychological disorders (Mushtaq et al., 2014; Yanguas et al., 2018). The COVID-19 brought a unique situation, in which workers can experience different types of loneliness that can be either chronic or transient (Young, 1982). There are workers who chronically experience loneliness, and do so in the COVID-19 (chronic loneliness); and there are other workers who do not experience loneliness in general but do experience in the COVID-19 (transient loneliness). For transient loneliness, it can be addressed with an online wellbeing meeting, where colleagues meet virtually to ‘check in’ each other’s wellbeing in the pandemic on a daily basis. Among university lecturers, this practice was reported helpful in reducing their transient loneliness (Kotera et al., in press). For chronic loneliness, re-appraisal of maladaptive social cognition was identified as the most effective intervention to reduce workplace loneliness in a comprehensive meta-analysis (Masi et al., 2011). In this regard, self-reflection may be recommended. Self-reflection can be done individually, reducing concerns about their mental health stigma.

Both types of loneliness can also be addressed in mindfulness interventions. A 2-week smartphone-based mindfulness intervention with 153 community adults showed positive results for reducing loneliness and increasing social contact (Lindsay et al., 2019). Similarly, a randomized controlled trial of an 8-week mindfulness training reported that the training significantly reduced loneliness (Zhang et al., 2018). Mindfulness helped workers to foster equanimity with loneliness, accepting the difficulties they were facing. Moreover, the positive effect of mindfulness training can also address the blurred work-home boundary—another COVID-19 work problem (Stiepan, 2020). Mindfulness reduced rumination about work when at home, and increased satisfaction with work and sleep quality (Crain et al., 2017). A mindfulness intervention at work can have a spill over effect in other contexts, such as home. These findings suggest that mindfulness-based interventions can be recommended to many organizations to address psychological issues related to remote work during COVID-19.

Mindfulness-based programs typically focus on the development of self-compassion. According to Neff (2003), the three main components of self-compassion are self-kindness, common humanity, and mindfulness. These oppose to self-judgment, isolation, and over-identification. Mindfulness Based Stress Reduction (MBSR) intervention has been shown to increase self-compassion (Shapiro et al., 2005). Consequently, an increase in self-compassion has positive effects on wellbeing because it makes the person feel connected, cared for, and calm (Gilbert, 2005). Examples of applications are the Compassion Focus Therapy (Gilbert, 2009), the Mindfulness for Health (Penman & Burch, 2013) and the Mindful Self-Compassion (MSC; Neff & Germer, 2013).

Finally, although a suggestion for compassion and mindfulness interventions might not be considered original, its value cannot be neglected. In fact, Galea (2020) has made an appeal for efforts in addressing the COVID-19 crisis to focus on compassion, arguing that its impact can go beyond physical and mental health, but also reduce inequalities widened by the pandemic by increasing individual’s resilience. Even though mental health issues have been a concern during the pandemic, efforts to address the negative psychological impacts on employees forced to work from home are still incipient.
